# Characterization, antibacterial properties, and application of gelatin films reinforced with smectite and illite clays for cherry fruit preservation

**DOI:** 10.1016/j.fochx.2025.102629

**Published:** 2025-06-04

**Authors:** Nermine Sayah, Nasir A. Ibrahim, Walid Elfalleh, Nacim Zouari, Noureddine Hamdi, Mourad Jridi

**Affiliations:** a Laboratory of Functional Physiology and Valorization of Bio-Resources (LR17ES27), Higher Institute of Biotechnology of Beja (ISBB), University of Jendouba, 9000, Beja, Tunisia; b Department of Biology, College of Science, Imam Mohammad Ibn Saud Islamic University (IMSIU), Riyadh 11623, Saudi Arabia; c Laboratory of Biodiversity, Molecules, and Applications (BMA) – LR22ES02, Higher Institute of Applied Biology of Medenine (ISBAM), Medenine, University of Gabes, Tunisia; d Higher Institute of the Sciences and Techniques of Waters of Gabes, University of Gabes, Tunisia

**Keywords:** Gelatin, Smectite, Illite, Structural and thermal properties, Wettability, Antibacterial and antioxidant activities

## Abstract

This study investigates the development of biodegradable composite films (F) based on gelatin (G), reinforced with smectite (Sm) and illite (IL) clays as fillers within the biopolymer matrix. A comprehensive set of characterization techniques was employed to evaluate the structural, thermal, mechanical, and barrier properties of the films. Mechanical testing revealed that incorporating 5 % clay significantly enhanced TS while reducing EAB compared to the control. Notably, the gelatin-illite film (GF-IL5%) exhibited superior mechanical stability and lower deformability compared to the gelatin-smectite counterpart (GF-Sm5%). Contact angle measurements indicated increased surface hydrophobicity, consistent with reduced WVP, suggesting improved barrier functionality. Additionally, the films showed enhanced antibacterial and antioxidant activities. Biodegradability tests and preservation trials on cherry fruits demonstrated that GF-Sm5% and GF-IL5% films contribute to prolong fruit freshness and microbial stability. These findings highlight the potential of Sm- and IL-reinforced gelatin films as high-performance materials for food preservation applications.

## Highlights

• GF-IL5% films showed the highest thermal stability among all tested formulations.
• Clay-reinforced gelatin films exhibited enhanced surface hydrophobicity.
• Strong interactions observed between clay nanoparticles and the gelatin matrix.
• Antibacterial, antioxidant, and biodegradation properties were significantly improved.
• Clay–gelatin films enhanced microbial stability of cherries during storage.

## 1 Introduction

Nowadays, the world is combating the emergence of harmful and toxic waste on the planet. A significant interest has been devoted to preserve our planet from toxicity, through different ways including the research of ecofriendly, sustainable and low-cost packaging (Ebrahimi et al., 2022). Therefore, packaging plays a vital role in fostering innovative strategies to address environmental challenges, while simultaneously ensuring the preservation of food quality and freshness. Packaging based on biopolymers are gained from environment-friendly renewable sources such as lipids, proteins, and polysaccharides (Bae et al., 2009). Among proteins, gelatin has garnered significant attention due to its affordability, biodegradability, and widespread availability. Moreover, it exhibits excellent film-forming properties, making it a versatile raw material for producing food packaging films (Ebrahimi et al., 2022). Unfortunately, it possessed some inconveniences, such as non-efficient barrier against water vapor penetration and poor mechanical property. To enhance these properties and greatly ameliorate their commercial potential, recent researches used to prepare packaging based on gelatin mixed with other polymers such as starch (Fakhouri et al., 2013) or nanoparticles (Nur Amila Najwa et al., 2020). Among others blending gelatin polymer matrix with clay, as a filler material has attracted substantial attention of researches (Dai et al., 2022; Rangaraj et al., 2022).

In this context, nanoparticles of clays and clay minerals presented the most important minerals used by manufacturing and environmental industries thanks to their low-cost abundance in nature and their distinctive crystal structure, which make them unique with special properties, involving adsorption capacity, high surface area and surface electric charges (Jlassi et al., 2017). Clays are naturally containing inorganic minerals categorized into various groups and they are classified according to their chemical composition as well as physical properties (Wang et al., 2022a, 2022b). Regardless the clay class, all-natural soil nanoclays are dominated by phyllosilicates and regularly contain metal hydroxides and organic matter (Warr, 2022). Moreover, nanomaterials based on clay are characterized by a layered mineral silicate with layered structural units which are responsible for defining aspects of nanoclays. In fact, Padil et al. (2022) showed that clay discerns a key parameter displayed in the arrangement of clay particles in an organized pattern and displayed high cations exchange capacity, retention properties and a high hydrophobicity.

Current scientific studies proved that dispersion of clay in a polymer matrix present a great exfoliated state which has a notable influence on the crystallization process and structural changes (Baniasadi et al., 2021). In addition, the incorporation of clays into polymer matrix inhibited the permeation of water molecules, and optimized the barrier properties of nanocomposite films (Nath, Pal, & Sarkar, 2022). Among studied minerals of clay, Smectite and Illite are examples that can act as a reinforcing agent to improve permeability, structural and mechanical properties of films (Alexandre et al., 2009).

The selection of clays in this study is based on their abundance in Tunisia and non-toxicity. Illite and smectite were specifically chosen for their functional and structural properties that enhance food packaging, including high surface area, porosity, hydrophilicity, and thermal stability. Deshmukh et al. (2023) showed that clays improve polymer composite matrices by enhancing dispersion and surface characteristics. Consequently, clay-based packaging is increasingly used for its chemical, mechanical, thermal, barrier, and biodegradable properties. In the same context, Bangar et al. (2023) highlighted the growing use of smectite minerals like bentonite in packaging for their mechanical, thermal, and swelling capacities. Nath, Santhosh, et al. (2022) also emphasized clays' compatibility with polymers, cost-effectiveness, and functional benefits. Therefore, this study evaluates the mechanical, structural, wettability, thermal, and biological performance of smectite and illite in food packaging.

The primary objective of this study is to develop biodegradable films by combining gelatin with smectite and illite, incorporated at concentrations of 1, 3, and 5 %. The research focuses on formulating gelatin-based biopolymer films enhanced with these clays and evaluating their effects on thermal stability, mechanical strength, structural integrity, as well as antibacterial and antioxidant properties. These characteristics are important for potential food packaging applications. Thus, to assess their practical performance, the developed films were applied to preserve cherry fruits.

## 2 Materials and methods

### 2.1 Materials

Two types of nanoclays were used in this study smectite and illite, recollected from the region of Gabes situated on the Southeast of Tunisia, precisely from Jebel Haidoudi (Aleg formation). Commercial gelatin from bovine was bought from Sigma Chemical Co. (St. Louis, Mo., U.S.A.). Anhydrous glycerol (LobaChemie, 98 % purity, India), employed as plasticizer, was obtained from Genencor International, USA.

### 2.2 Clay purification

Clay samples (smectite, illite) were sourced from the upper layers of the Aleg Formation (92) and subjected to a multi-step purification process to ensure optimal mineral refinement. The purification of clays was determined as described by Yahya et al. (2021). Initially, raw clay was oven-dried at 80 °C to remove excess moisture. To eliminate carbonate impurities, the dried clay was treated with 0.5 N HCl under constant stirring, allowing sufficient reaction time for carbonate dissolution. Subsequently, H₂O₂ treatment was applied to degrade and remove organic matter, ensuring minimal interference from naturally occurring organic residues. To enhance clay dispersion and carbonates removal, the samples were subjected to five successive washes with 2 M NaCl solution, promoting ionic exchange within the clay interlayers. Following the washing steps, the suspensions were subjected to centrifugation at 6000 rpm for 30 min, effectively separating purified clay fractions from residual contaminants. The resulting pellets were then dried at 60 °C for 24 h, following the protocols established by Churchman et al. (2006), stabilizing their structure while preventing agglomeration. The purified clay samples were subsequently designated as Sm (smectite) and Il (illite) for further characterization.

### 2.3 Films preparation

Nanocomposite films were prepared by dissol*v*ing 4 % (*w*/*v*) of gelatin in distilled water at 50 °C for 30 min using heating magnetic. Simultaneously, 1, 3 and 5 % of Sm and Il was dispersed at room temperature in distilled water and then incorporated into the gelatin solution (*w*/w of gelatin). Later, glycerol, accounting for 15 % (w/w of the polymers), was used as a plasticizer, and the mixtures were gently stirred for 30 min. Subsequently, 25 mL of each film-forming solution (FFS) was cast into 12 × 12 cm Petri dishes and left to dry in a ventilated climatic chamber set at 30 °C with a controlled relative humidity (RH) of 50 % for 48 h. The dried films were carefully removed and equilibrated for three weeks at 50 % RH before undergoing analysis.

GF indicated control gelatin film, GF-Sm1%, GF-Sm3% and GF-Sm5 % indicated gelatin films added by 1, 3 and 5 % of smectite, respectively. GF-Il1%, GF-Il3% and GF-Il5 % indicated gelatin films added by 1, 3 and 5% of illite, respectively.

### 2.4 Films characterization

#### 2.4.1 Thickness

The thickness of the films was measured using a digital micrometer (PosiTector 6000, DeFelsko Corporation, USA). For each sample, measurements were taken at ten random locations, and the average thickness value was calculated. The mean thickness was subsequently used in the evaluation of mechanical properties, water vapor permeability (WVP), and transparency.

#### 2.4.2 Water solubility

The solubility of the films (WS) was evaluated based on the method described by Khoshkalampour et al. (2023), with minor modifications. Square film samples (20 mm × 20 mm) were initially oven-dried at 105 °C for 8 h until a constant weight was achieved. The initial weight of the dried films was recorded as (mi). Subsequently, the samples were immersed in 50 mL of distilled water and agitated at 150 rpm for 6 h at 25 °C. The solution was then filtered, and the insoluble residue was collected and dried at 105 °C for 24 h to reach a constant weight. The final weight of the dry residue was recorded as (mf), and the water solubility (WS) of the films was calculated using the following formula:

(1) $$\mathrm{WS}\;\left(\%\right)=\frac{\mathrm{mi}-\mathrm{mf}}{\mathrm{mi}}\times 100$$

#### 2.4.3 Color, light transmission and transparency

The color parameters of the clay/gelatin nanocomposite films were analyzed using a colorimeter CR-200 (Minolta, Japan). The color of the films was characterized by L* (lightness/brightness), a* (redness/greenness), and b* (yellowness/blueness) values. Additionally, the color saturation (chroma, C*), the total color difference (∆E*), and the hue angle (h*) of the gelatin and clay/gelatin films were calculated using the respective formulas.

(2) $$C*=\sqrt{a{*}^{2}+b{*}^{2}}$$

(3) $$h^{*}=\arctan\;\left(b^{*}/a^{*}\right)$$

(4) $$∆E*={\left[{\left(∆L*\right)}^{2}+{\left(∆a*\right)}^{2}+{\left(∆b*\right)}^{2}\right]}^{1/2}$$

where ∆L*, ∆a* and ∆b* are the differences between the color parameter of the control film and those added with smectite and Illite. A standard white plate (L^0^ = 97.5; a^0^ = − 0.1 and b^0^ = 2.3) was utilized to adjust the Chromameter.

The barrier properties of films against ultraviolet (UV) and visible light were recorded employing spectrophotometer (SAFAS Monaco, UVmc) across a wavelength range of 200 to 800 nm. The transparency value was calculated using the following equation.

(5) $$\text{Transparency value}=-\log\;T_{600}/e$$

where T600 is the fractional transmittance at 600 nm and e is the film thickness (mm).

#### 2.4.4 Mechanical properties

The mechanical properties of the clay/gelatin nanocomposite films were evaluated using a texture analyzer (TA. HD Plus model, Stable Micro Systems, UK) equipped with A/MTG tensile grips and a 300 N load cell, following the ASTM-D882 method (ASTM, 1985) at 25 °C and 50 % relative humidity (RH).

Rectangular film samples (4 × 2 cm) were precisely cut using a standard precision cutter (Thwing-Albert JDC Precision Sample Cutter, USA) to ensure uniform dimensions. The films were mounted between the grips of the analyzer and stretched at a crosshead speed of 50 mm/min until they fractured. Tensile strength (TS, MPa) and elongation at break (EAB, %) were calculated based on the force and deformation data recorded by the software. Each analysis was repeated six times per sample to ensure accuracy and consistency.

#### 2.4.5 X-ray diffraction (XRD)

X-ray diffractions were used to identify the different elements present in the matrix clay-gelatin and to investigate the preferred orientation of gelatin in the presence of the clay. X-ray diffractograms of clay/gelatin nanocomposite films were obtained using an X-ray diffractometer model X'PERT Pro MPD PANALYTICAL. Samples (2 × 2 cm) were fixed in an aluminum frame and scanned at 25 °C, over a diffraction angle 2θfrom 5° to 90°, at 30 kV and 10 mA with a scan rate of 2°/min.

#### 2.4.6 Fourier transform infrared spectroscopy (FTIR)

The infrared absorption spectra of clay/gelatin nanocomposites films were acquired using a PerkinElmer Spectrum infrared spectrometer (Nicolet, Ettlingen, Germany) equipped with an attenuated total reflection (ATR) accessory. The films were analyzed with 32 scans per minute at a resolution of 4 cm^−1^, covering the wavenumber range from 600 to 4000 cm^−1^.

#### 2.4.7 Contact angle measurements and surface properties

The surface properties of food packaging films, including surface tension and wettability, were evaluated using the sessile drop method as described by Karbowiak et al. (2006). A goniometer (Krüss Drop Shape Analyzer, Germany) equipped with image analysis software (Krüss Advance, version 1.4.1.2) was used for the measurements. This technique involves depositing a liquid droplet onto the material's surface and determining its contact angle.

For the analysis, the films were mounted onto glass slides placed on a movable stage connected to the contact angle analyzer. A droplet (2 μL) of one of four liquids (water, ethylene glycol, glycerol, or diiodomethane) was carefully dispensed onto the film surface using a syringe. The camera recorded images of the droplet's contact angle on both sides and tracked any changes over time. The contact angles (ɵ) for the four liquid droplets were measured six times for each film sample, and the results were averaged to ensure accuracy and reliability.

The film surface tensionγ_S_ and its dispersive γ_L_^D^ and polar γ_L_^P^ components as well as its critical surface tension γ_c_were estimated conforming to the following formula:

(6) $$\gamma_{S}=\gamma^{P}+\gamma^{D}$$

(7) $$\gamma_{\mathrm{Li}}\;\left(1+\cos\theta i\right)=2\;{\left[{\left({\gamma_{S}}^{D}\times {\gamma_{\mathrm{Li}}}^{D}\right)}^{1/2}+{\gamma_{S}}^{P}\times {\gamma_{\mathrm{Li}}}^{P}\right)}^{1/2}]$$

where L and S are relevant to the liquid and solid film surface, respectively. The surface tension γ_L_of the tested liquids: (water (γ_L_ = 72.8 mN/m; γ^*P*^ = 51mN/m; γ^D^ = 21.8 mN/m), ethylene glycol (γ_L_ = 47.7 mN/m; γ^*P*^ = 21.3 mN/m; γ^D^ = 26.4 mN/m), diiodomethane (γ_L_ = 50.8mN/m; γ^*P*^ = 2.3 m N/m; γ^D^ = 48.5 mN/m) and glycerol (γ_L_ = 63.4 mN/m; γ^*P*^ = 26.4 mN/m; γ^D^ = 37 mN/m)) are known at 25 °C. The critical surface tension of wetting (γ_C_) is determined by extrapolating to the value of (γ_L_) of each liquid corresponding to cos (θ) = 1 (Halper et al., 1972).

#### 2.4.8 Water vapor permeability

Water vapor permeability (WVP) refers to a material's capacity to allow water vapor to pass through a unit area over a defined period, influenced by factors such as partial pressure differential and thickness. In this study, WVP was assessed gravimetrically using a modified ASTM E96–80 (1980) standard method, specifically adapted for edible materials. The experimental conditions included a temperature of 25 °C and 50 % RH.

The procedure involved cutting and sizing the films, which were then placed between two Teflon rings positioned on top of a glass cell filled with distilled water (100 % RH). These assemblies were incubated in a climatic chamber (KBF 240 Binder, France) maintained at 50 % RH and 25 °C. The weight of each cell was recorded daily for 21 days to track water vapor transmission. Four samples were utilized for WVP measurements. The WVP (10^−10^ g m^−1^ s^−1^ Pa^−1^) of all films was calculated as follows:

(8) $$\mathrm{WVP}=\frac{∆w.e}{A.∆t.\left(p1-p2\right)}$$

where ∆w is the weights variation (g); e is the film thickness (m); A is the exposed area of each film (9.08 10^−4^ m^2^); ∆t is the time of weight variation (s); ∆P is the vapor partial pressure differential across the film (Pa).

#### 2.4.9 Thermal properties

##### 2.4.9.1 Differential scanning calorimetry (DSC)

To examine the interaction between clay nanoparticles and gelatin, a DSC analysis was performed as previously described by Sayah et al. (2024). Prior to the analysis, the samples were conditioned for 72 h at 25 °C and 0 % RH to achieve a dried film state. The films were analyzed using a DSC Q20 device, controlled by TA Universal Analysis software (TA Instruments Universal Analysis 2000; version 4.5 A), under a nitrogen flow rate of 25 mL/min. Approximately 5 mg of each sample was sealed in DSC aluminum pans (PerkinElmer) and subjected to heating at a rate of 10 °C/min across two cycles. The temperature range for the DSC scan spanned from 25 to 150 °C. During the first scan, the samples were stabilized at 25 °C, cooled to −50 °C, held isothermally for 3 min, heated up to 150 °C, and subsequently cooled back down to 25 °C. An empty pan was used as a reference throughout the analysis.

##### 2.4.9.2 Thermogravimetric analysis (TGA)

TGA consists on weighing the sample by a precision balance when it is heated and cooled using a SDT Q600 (TA Instruments). Samples were implanted in pans with an initial weight of 8–10 mg under a controlled dry nitrogen flow of 20 mL/min. The variation of weight of the film is monitored as a function of temperature as the samples are maintained to a controlled temperature program in a controlled atmosphere, within the range 20–900 °C at a heating rate of 20 °C/min.

#### 2.4.10 Scanning electron microscopy

Samples' microstructure was observed using a MEB. A diameter of 40 μm was designated and accelerating voltage of 15 keV to ensure the calibration. The working distance was set at WD = 8.4 mm and clay/gelatin nanocomposites films were attached on platinum stubs for viewing.

### 2.5 Biological activities of smectite-gelatin and illite-gelatin films

#### 2.5.1 Antimicrobial activity of clays-gelatin films

##### 2.5.1.1 Microbial strains

The antibacterial activity of illite or smectite and gelatin films was assessed against gram positive *Staphylococcus aureus* (ATCC 25923), *Bacillus cereus* and gram-negative *Pseudomonas bacteria* and *Escherichia coli* (ATCC 25922), using agar diffusion method.

##### 2.5.1.2 Agar diffusion method

The antibacterial activity of smectite-gelatin and illite-gelatin films was determined using the method described by Berghe et al. (1991). Each culture suspensions, containing 200 μL of microorganisms (with a concentration of 10^6^ colony-forming units (CFU/mL), determined by absorbance at 600 nm) were evenly spread on Luria-Bertani (LB) agar plates, already caste in petri dishes.

Firstly, 60 μL of dispersed smectite-gelatin or illite-gelatin films at a concentration of 10 mg/mL was carefully placed in 6 mm-diameter wells, which were fixed in the agar layer. Gentamycin at a concentration of 30 μg/well was used as a positive control for the tested bacteria. To ensure the diffusion of smectite-gelatin or illite-gelatin dispersion, the petri dishes were kept at 4 °C for 1 h. Following to this, petri dishes were incubated for 24 h at 37 °C. The effectiveness of antibacterial activity was estimated by measuring the diameter in mm of the inhibition zones in mL, which announce the extent of growth inhibition caused by these films or the positive control (gentamycin).

#### 2.5.2 Evaluation of antioxidant activities

To evaluate antioxidant activities of films, two methods were employed:

***Free radical scavenging activity on 1,1-diphenyl-2-picrylhydrazyl (DPPH)*:** The DPPH•-radical scavenging activity was evaluated as described by Bersuder et al. (1998). Firstly, 500 μL of composite films solution were mixed with 375 μL of ethanol solution and 125 μL of 0.02 % DPPH. The mixture was incubated for 60 min in the dark at room temperature (25 °C) and the reduction of DPPH was measured at 517 nm. The test was executed in triplicate and the DPPH-radical scavenging activity was determined as follows:

(9) $$\text{Scavenging activity}\;\left(\%\right)=\left[\left({\mathrm{OD}}_{C}\;{\mathrm{OD}}_{S}\right)/{\mathrm{OD}}_{C}\right]\times 100$$

where OD_C_, and OD_S_ reveal the absorbance's of the control and the sample reaction tubes, respectively.

***Metal-chelating activity***: The chelating activity of smectite-gelatin and illite-gelatin films toward ferrous ion (Fe^2+^) was evaluated as described by Decker and Welch (1990). One mL of each dispersed films at different concentrations (1, 2, 5 mg/mL) were mixed with 3.7 mL of distilled water. Then, the addition of 0.1 mL of 2 mM FeCl_2_, 4H_2_O and 0.2 mL of 5 mM 3-(2-pyridyl)-5,6-bis (4-phenyl-sulfonic acid)-1,2,4-triazine (ferrozine) was done. Thereafter, the obtained mixture was incubated for 20 min at 25 °C. The absorbance of each sample was then read at 562 nm. The blank was conducted in the same manner except that distilled water was used instead of the sample. EDTA was used as reference. The chelating activity (%) was calculated as follows:

(10) $$\text{Metal chelating activity}\;\left(\%\right)=1-\frac{\mathrm{OD}\;\text{of sample}}{\mathrm{OD}\;\text{of blank}}\times 100$$

### 2.6 Biodegradation test

The biodegradation test was investigated according to Qi et al. (2021) with slight modifications. Color and film surface integrity were visually recorded. At first, rectangular film samples (2 × 2 cm) were cut using a standard precision cutter (Thwing-Albert JDC Precision Sample Cutter, USA) to ensure uniform dimensions. The films were entered in ground. Each day the film was retrieval from the soil, the soil fixed to the film surface was removed by distilled water and dried at room temperature. Thus, film weight loss (FWL) was employed as an indicator of film degradation and was calculated as the following formula:

(11) $$\mathrm{FWL}\;\left(\%\right)=\left(m_{0}-m_{1}\right)/m_{0}\times 100$$

where m_0_ (g) and m_1_ (g) is the dry weight of films before and after burial. These measurements were examined only 10 days.

### 2.7 Application for cherry preservation

#### 2.7.1 Cherry fruits coating

A batch of fresh cherry were harvested from Makther, Tunisia, were delivered by cold chain transport (4 °C with 70 % humidity) and diseased cherry were removed. Selecting moderate size and analogous maturity cherry and then dipped for 3 min in 1 % sodium hypochlorite solution for surface disinfection, washed with distilled water and air dried. After drying, the fruits were immersed into different coating solutions (GF, GF-Sm5%, or GF-IL5%) for 2 min. After immersion the solution was allowed to drip off, the fruits were then subjected to air drying at room temperature (20 °C). Fruits without any treatment were dipped in distilled water and designated as control. Finally, samples were packaged in polyethylene pouches (64 μm thickness) and stored under refrigeration at 4 ± 1 °C for the following tests. Both coated and control fruits were analyzed up to 12 days of storage at an interval of 4 days.

#### 2.7.2 Fruits characterization

##### 2.7.2.1 Weight loss

Weight loss was calculated as loss in weight of the cherry fruits during storage and the values were reported as percentage. Five fruits per replication were taken, and measurements for each group were performed in triplicate.

##### 2.7.2.2 Titratable acidity

Titratable acidity (TA) was measured by titrating 10 mL of already prepared cherry juice with 0.1 N NaOH. Phenolphthalein was used as color indicator and results were expressed as % malic acid. Measurements were performed in triplicate.

(12) $$\mathrm{TA}=\frac{(\left(V\;\left(\text{NaOH}\right)\times 0.1\times 0.064\right)}{m}\times 100$$

where V (NaOH) is the volume (mL) of titrated NaOH, 0.1 is the molarity of NaOH solution, 0.064 is the conversion factor for citric acid, and m is the mass of the aliquot sample.

##### 2.7.2.3 Antioxidant activity

Fruit pulp 1 g was homogenized with methanol and the extract was centrifuged at 7000 rpm for 10 min. After centrifugation the mixture was filtrated, and clear supernatant was collected. Then, the DPPH radical scavenging and metal chelatig activities of cherry fruit during storage were measured as previously described in the section 2.5.2

##### 2.7.2.4 Color

Cherry surface color values were measured directly using hunter lab colorimeter (USA Virginia Hunter Lab Colorimeter). Five fruits per replication from each coated and uncoated fruit were evaluated.

##### 2.7.2.5 Microbiological assessment

Cherry fruit (10 g) was mixed with 90 mL of sterile saline solution and then homogenized for 10 min. After homogenization, 1 mL of each sample was transferred to plate count agar (PCA) containing petri dishes and incubated at 5 °C for 7 days to determine the psychrophilic bacterial count. For determination of mold and yeasts count, the sample was transferred to petri dishes containing chloramphenicol glucose agar (CGA) and potato dextrose agar (PDA). Serial 10 dilutions were made in each treatment. Finally, petri plates were incubated at 30 °C for 3 days. Analysis was performed in replicates; results were expressed in log10 CFU/g.

### 2.8 Statistical analysis

All analytical results were presented as mean values accompanied by their respective standard deviations (SD). A one-way analysis of variance (ANOVA) was conducted using SPSS software (version 18.0). Statistical significance was determined at a threshold of *p* < 0.05.

## 3 Results and discussion

### 3.1 Film thickness and solubility

Thickness is a key attribute of thin films, closely influencing their mechanical, optical, and barrier properties. As presented in Table 1, the measured thickness values for both gelatin films and clay/gelatin composite films indicate that the addition of clay to the gelatin matrix did not significantly alter the thickness of the films (*p* > 0.05). Furthermore, the films exhibited flexibility and were easily detached from the Petri dishes, maintaining a uniform and homogeneous appearance. Moreover, the WS of all films presented in Table 1**.** Control film (Gelatin at 4 %) showed the highest solubility (61 %) compared to other composite films (*p* < 0.05). With increasing Smectite/Illite content, solubility significantly decreased resulting in more resistant films after the addition of clay. Indeed, the minimum solubility was recorded at 39 % and 34 % for GF-Sm5% and GF-IL5%, respectively. This result explained the high hydrophobic nature of nanoclay (Smectite/Illite) particles. These results are similar to those obtained by Farahnaky et al. (2014), how reported that nanoclay particles acted as a barrier against water molecules movements and minimized effective contact between solvent molecules and gelatin. The authors reported a decline in the cloisite concentration reinforced the water absorption of gelatin–nanocomposite films which reduced the water uptake of blended films.

**Table 1:** Thickness, solubility, thermal, water vapor permeability and mechanical properties of films.

	Thickness (μm)	TS (MPa)	EAB (%)	WS (%)	Tg (°C)	WVP (10^−10^ g m^−1^ s^−1^ Pa^−1^)
GF	58.0 ± 2.0 ^a^	53.1 ± 4.27 ^c^	5.79 ± 0.45 ^a^	61.0 ± 0.25 ^a^	18.02 ± 0.20 ^a^	2.63 ± 0.28 ^a^
GF-Sm1 %	52.0 ± 4.0 ^b^	50.55 ± 3.73 ^c^	2.42 ± 0.99 ^b^	53.9 ± 0.65 ^b^	17.38 ± 0.28 ^a^	2.47 ± 0.87 ^a^
GF-Sm3 %	53.0 ± 3.0 ^b^	55.42 ± 6.46 ^bc^	2.83 ± 0.30 ^b^	43.9 ± 1.5 ^c^	22.31 ± 0.10 ^a^	2.36 ± 0.28 ^a^
GF-Sm5 %	60.0 ± 2.0 ^a^	62.88 ± 3.71 ^b^	2.22 ± 0.28 ^b^	38.9 ± 0.35 ^d^	26.41 ± 0.16 ^a^	1.76 ± 0.19 ^a^
GF-IL1 %	43.0 ± 3.0 ^c^	60.96 ± 3.62 ^b^	2.41 ± 0.24 ^b^	43.9 ± 0.95 ^c^	25.76 ± 0.42 ^a^	2.52 ± 0.45 ^a^
GF-IL3 %	41.0 ± 3.0 ^c^	65.07 ± 2.88 ^ab^	2.51 ± 0.35 ^b^	37.7 ± 0.45 ^d^	24.44 ± 0.11 ^a^	2.18 ± 0.16 ^a^
GF-IL5 %	46.0 ± 2.0 ^c^	68.63 ± 1.0 ^a^	2.73 ± 0.15^b^	33.8 ± 0.50 ^d^	27.57 ± 0.15 ^a^	1.80 ± 0.28 ^a^

**TS:** Tensile strength; **EAB:** Elongation at break. GF indicates gelatin film. GF-Sm1 %, GF-Sm3 %, GF-Sm5 %, GF-IL 1 %, GF-IL3 %, GF-IL5 % indicate gelatin film added by 1 %, 3 % and 5 % of smectite and illite, respectively. All measurements were performed at 25 °C and RH = 50 %. ^a,b,c^ Different letters in the same column indicate significant difference (*p* < 0.05).

### 3.2 Mechanical properties of composite films

The mechanical strength, resistance and flexibility of films are critical parameters to ensure the quality of food packaging. EAB and TS of composite clay/gelatin films are displayed in Table 1. TS and EAB of the control gelatin film were about 53.1 MPa and 5.79 %, respectively. The incorporation of Sm and Il clays at varying concentrations into the gelatin matrix led to a notable enhancement in TS values. Specifically, the results showed that TS increased progressively with higher clay concentrations, reaching 62.88 MPa for GF-Sm5% and 68.63 MPa for GF-IL5%. This improvement in TS can be attributed to the formation of new interactions between the clay particles and the gelatin matrix (Li et al., 2015) which increase the rigidity of films. The decrease of EAB values can be attributed to a decrease in gelatin chain mobility which lowered significantly the film flexibility (Rangaraj et al., 2022). In the same context, Karimi et al., (2013) demonstrated that gelatin/laponite coacervates could establish a robust interconnected gel-like network structure, driven by electrostatic interactions between the gelatin molecules and the laponite platelets.

Additionally, gelatin film exhibited the highest elongation at break (EAB) value (*p* < 0.05), which diminished as the clay concentration increased, reaching 2.22 % for GF-Sm5% and 2.73 % for GF-IL5%. However, no significant changes in EAB were observed at other clay/gelatin ratios (*p* > 0.05).

The TS and EAB values obtained in this study were compared with previous findings to assess the effect of various nanoclays on gelatin-based films. Valencia et al. (2016) reported that incorporating laponite into gelatin films enhanced their TS and EAB due to electrostatic interactions between negatively charged amino acid groups in gelatin and the positively charged layers of laponite, which strengthened the polymer matrix. In contrast, Nur Amila Najwa et al. (2020) observed lower TS values in gelatin films containing kaolinite or modified kaolinite compared to those with smectite or illite. This reduction was attributed to the elasticity of gelatin and the presence of strong peptide linkers, which limited the interaction between kaolinite and the gelatin matrix. Furthermore, the decreased TS and EAB values were associated with weakened interactions between hydrophobic silver compounds and hydrophilic gelatin chains, as well as restricted chain mobility due to kaolin dispersion. Similarly, Rangaraj et al. (2022) reported that increased TS in gelatin films was related to effective interfacial stress transfer facilitated by interactions between silver-sepiolite particles and the NH groups of gelatin, although this was accompanied by reduced EAB due to inhibited polymer mobility. Compared to these studies, the TS values in the present work are higher than those reported by Rangaraj et al., while the EAB values are lower, suggesting a more rigid but stronger film structure.

### 3.3 Water vapor permeability of composite films

Food packaging should act as a barrier against moisture to prevent spoilage caused by microorganisms' development, resulting in longer shelf life. In this study, the WVP of GF-Sm and GF-Il films was estimated at 50 % RH differential. Table 1 shows the results relative to WVP of different films. A decrease of WVP in clay/gelatin films 2.63 ± 0.28 10^−10^ g/m.s.Pa for control film and 1.76 ± 0.19 10^−10^ g/m.s.Pa and 1.80 ± 0.28 10^−10^ g/m.s.Pa for GF-Sm5% and GF-IL5%, respectively. In this sense, Nur Amila Najwa et al. (2020) reported that kaolin and silver addition effectively prevent the penetration of water vapor by creating a complex path against water diffusion across the polymer matrix. Control film's tensile strength (53.1 MPa), elongation at break (5.79 %), WS (61.0 %), Tg (18.02 °C) and WVP (2.63 10^−10^ g m^−1^ s^−1^ Pa^−1^) are consistent with reported values for pure gelatin films under similar preparation conditions (Liu et al., 2019; Said & Sarbon, 2022).

### 3.4 Thermal properties of composite films

#### 3.4.1 Differential scanning calorimetry

The DSC spectra of Smectite-gelatin and Illite-gelatin nanocomposite films, as presented in Table 1, exhibited characteristics typical of partially crystalline materials. A single glass transition temperature (Tg) was observed for all samples, ranging between 18 and 28 °C, indicating good compatibility between the clay and gelatin components. Notably, the Tg values did not significantly exceed the room temperature of 25 °C, reinforcing the amorphous nature of the films.

The control film displayed a Tg of approximately 18 °C, which was lower than the Tg values recorded for GF-Sm5 % (26.41 °C) and GF-IL5 % (27.57 °C). The incorporation of Sm and Il at concentrations ranging from 1 to 5% within the gelatin matrix led to an increase in Tg, signifying enhanced thermal stability and suggesting that the clays function as heat barriers during the polymer's thermal degradation, as highlighted by Zhu et al. (2019).

Additionally, a positive correlation was observed between Tg and TS values of the nanocomposite films, with higher Tg values corresponding to films exhibiting the greatest TS measurements. López-Angulo et al. (2020) noted a decrease of Tg values comparing with control film which can be explained by a feeble attraction between negative charge of surface layers of laponite and positive charge of amino acids of gelatin chains.

#### 3.4.2 Thermogravimetric analysis

**Fig. 1:**
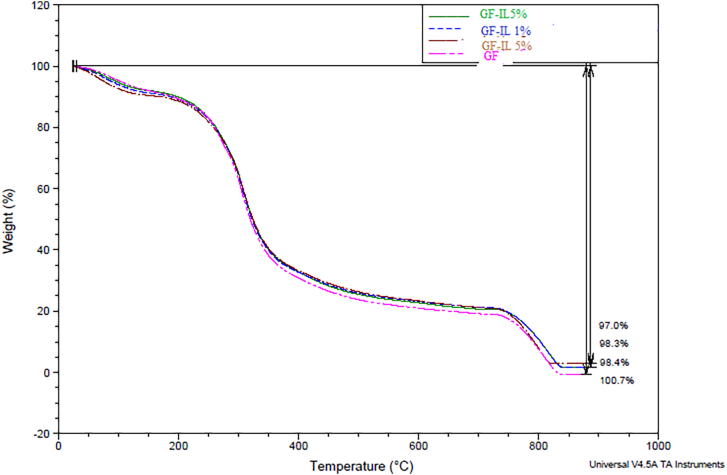
Thermogravimetric analysis scans of gelatin and nanocomposite films. (a) Smectite/gelatin nanocomposite films and (b) Illite/gelatin nanocomposites films.

TGA was employed to evaluate the thermal stability of the films over a broad temperature range. As shown in Fig. 1, the TGA curves showed distinct degradation phases. The first phase, occurring near 100 °C, corresponds to a minor mass loss attributed to the evaporation of physically adsorbed water and interlayer water present in the silicates (Rat et al., 2023). A more substantial weight loss was observed between 200 and 320 °C, mainly associated with the thermal degradation of gelatin's amino acid components, while the clay fillers remained thermally stable during this stage (López-Angulo et al., 2020). At higher temperatures (400 to 750 °C), further degradation was detected, which can be explained by the volatilization of glycerol and partial decomposition of the clay structures (López-Angulo et al., 2020). Notably, the control GF showed complete mass loss by the end of the analysis, whereas the GF-IL5% retained approximately 3 % of its original mass, indicating improved thermal stability conferred by the clay incorporation.

### 3.5 Light transmission and opacity of films

Optical properties are important to be assessed in packaging as they provide physical and biochemical protection during the shipping process. Also, edible films with increased UV light absorbance are interesting as they can inhibit food peroxidation (Ye et al., 2022). Transmission of UV and visible light at selected wavelengths (200-800 nm) of different samples are shown in Table 2. The incorporation of clay into gelatin matrix significantly decreased the transmittance values in the UV and visible regions between 300 and 800 nm was detected. The transparency results showed that the control film exhibited the highest lightness, whereas the incorporation of clay caused a slight increase in the darkness of the composite films. This effect can be attributed to the colored particles within the clays, which absorb light in the visible spectrum, thereby influencing the opacity of the composite films. However, Valencia et al. (2016) demonstrated that nanocomposite films opacity was not affected by laponite addition.

**Table 2:** Light transmission and opacity of films.

Wavenumbers (cm^−1^)	200	280	300	400	500	600	700	800	Transparency
GF	0.01	0.01	4.45	34.30	40.50	43.80	46.92	48.30	6.18 ± 0.3 ^g^
GF-Sm1%	0.01	0.01	2.60	24.70	30.40	33.50	35.80	37.70	9.13 ± 0.2 ^d^
GF-Sm3 %	0.01	0.01	2.80	26.50	32.85	36.10	39.23	41.20	8.35 ± 0.1 ^f^
GF-Sm5 %	0.01	0.01	1.45	10.00	12.60	14.10	15.50	16.90	14.18 ± 0.16 ^a^
GF-IL1%	0.01	0.01	1.60	29.10	38.45	42.20	45.20	47.30	8.71 ± 0.17 ^e^
GF-IL3%	0.01	0.01	2.00	26.35	33.10	36.30	38.70	40.35	10.73 ± 0.15 ^c^
GF-IL5%	0.01	0.01	1.00	18.20	26.15	30.40	33.75	36.25	11.24 ± 0.1 ^b^

GF indicates gelatin film.GF-Sm1%, GF-Sm3%, GF-Sm5%, GF-IL 1 %, GF-IL3 %, GF-IL5 % indicate gelatin film added by 1 %, 3 % and 5 % of smectite and illite, respectively. All measurements were performed at 25 °C and RH = 50 %. ^a,b,c^ Different letters in the same column indicate significant difference (*p* < 0.05).

Color is a critical quality parameter often evaluated by consumers as it directly relates to the visual appeal of food products and influences consumer perception (Salem et al., 2021). The color parameters (L∗, a∗, b∗, C*, h*, and ΔE*) of the films were presented in Table 3**.** The incorporation of clays into the gelatin matrix resulted in an increase in L* and b* values, accompanied by a decrease in a* values. Specifically, the addition of clay enhanced the yellowish tone (b*), increasing from −0.07 in the control film to 1.69 in GF-Sm5% and 2.48 in GF-IL3%.

**Table 3:** Color parameters of films.

	L*	a*	b*	C*	h*	∆E
GF	39.04 ± 0.35 ^d^	−0.22 ± 0.02 ^b^	−0.07 ± 0.01 ^f^	0.23 ± 0.01 ^a^	98.87 ± 0.09 ^a^	–
GF-Sm1 %	40.55 ± 0.16 ^c^	−0.08 ± 0.01 ^a^	0.47 ± 0.05 ^d^	0.47 ± 0.05 ^a^	99.38 ± 0.07 ^a^	0.95 ± 0.07 ^d^
GF-Sm3%	40.76 ± 0.55 ^c^	−0.24 ± 0.01 ^b^	0.43 ± 0.01 ^d^	0.49 ± 0.02 ^a^	118.97 ± 0.02 ^a^	0.86 ± 0.05 ^d^
GF-Sm5 %	40.23 ± 0.15 ^c^	−0.38 ± 0.07 ^bc^	1.69 ± 0.07 ^c^	1.73 ± 0.02 ^c^	102.55 ± 0.95 ^a^	1.76 ± 0.01 ^c^
GF-IL1%	37.3 ± 0.49 ^e^	−0.45 ± 0.01 ^c^	0.17 ± 0.01 ^e^	0.48 ± 0.02 ^a^	159.33 ± 0.94 ^a^	0.50 ± 0.01 ^e^
GF-IL3%	41.77 ± 0.26 ^b^	−1.07 ± 0.07 ^e^	2.48 ± 0.50 ^b^	2.7 ± 0.10 ^b^	113.29 ± 0.67 ^a^	2.04 ± 0.01 ^b^
GF-IL5%	49.60 ± 0.33 ^a^	−1.56 ± 0.09 ^d^	6.67 ± 0.28 ^a^	6.85 ± 0.09 ^a^	103.16 ± 0.66 ^a^	3.46 ± 0.08 ^a^

GF indicates gelatin film. GF-Sm1%, GF-Sm3%, GF-Sm5%, GF-IL 1 %, GF-IL3 %, GF-IL5 % indicate gelatin film added by 1 %, 3 % and 5 % of smectite and illite, respectively. All measurements were performed at 25 °C and RH = 50 %. ^a,b,c^ Different letters in the same column indicate significant difference (*p* < 0.05).

Additionally, blending clay with gelatin raised the L* value from 39.04 in the control GF to 40.23 in GF-Sm5%. The color saturation (C*) values remained below 10, indicating the intensity and brilliance of the color in the clay/gelatin nanocomposite films. These findings are consistent with Farahnaky et al. (2014), who observed increased lightness and yellowness, alongside reduced redness, following cloisite addition.

Furthermore, the total color difference (ΔE) values increased with clay incorporation, with the highest recorded ΔE value of 3.46 ± 0.08 for GF-IL5%. These variations are primarily attributed to the inherent color of Sme and Il clays.

### 3.6 X-ray diffraction analysis

**Fig. 2:**
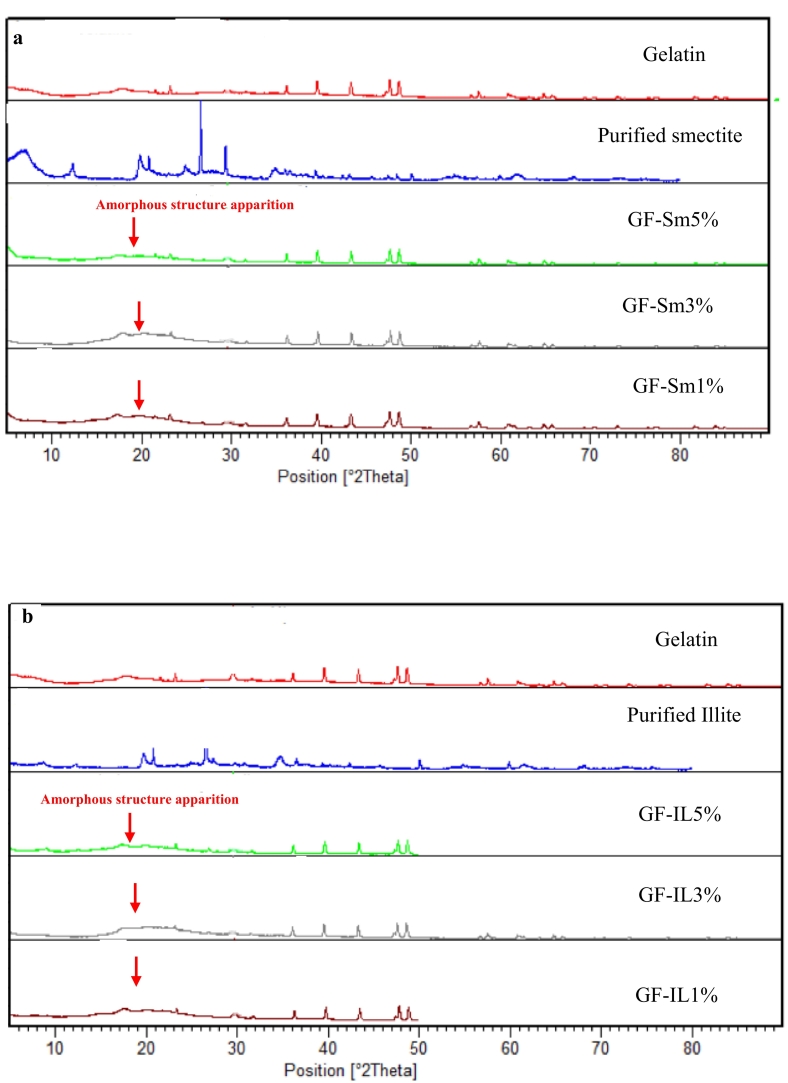
X-Ray diffraction of films; measurements were done at 25 °C and RH 50 %. (a)Smectite/gelatin nanocomposite films and (b) Illite/gelatin nanocomposites films.

XRD is widely used to investigate the structure and the crystallinity of clay-gelatin films. The XRD patterns of clay-gelatin films are illustrated in Fig. 2 (a, b). The diffractogram showed a similar feature with a dominant peak at 2θ = 30°, which can be assigned to quartz (Deon et al., 2022). X-ray of all nanocomposite films showed an insignificant diffraction peak of gelatin crystal, which proved the dispersion of nanoclay particles into the biopolymer matrix and demonstrated that the addition of clay decreased the crystallization of the gelatin (Rao, 2007). Additionally, XRD diffractograms of nanocomposite films exhibited some minor reflections due to the presence of multiple crystalline phases such as quartz and phyllosilicates.

The broad diffraction band observed at 2θ ≈ 30° was characteristic of the amorphous nature of gelatin and suggests interactions between gelatin chains and the illite-smectite clay layers. Additionally, the X-ray diffractograms showed that the basal reflection of the clays (d₀₀₁ = 8.5 Å at 2θ ≈ 6°) remained largely unchanged, indicating that the gelatin chains were dispersed within, rather than significantly expanding, the interlayer space of the smectite and illite. These results support the intercalation of gelatin molecules between silicate layers, which likely contributed to the observed increase in TS following clay incorporation. Similar findings were reported by Sudhakar et al. (2021), who reported that polymer intercalation into organoclay (modified montmorillonite) was evident from XRD analysis.

Additionally, these results demonstrated that after treating with polymers clays peaks were shifted in the intercalated nanocomposites which proved an increase of the basal interlayer spacing of clay by the intercalation of polymer (Zainuddin et al., 2010).

### 3.7 Fourier transform infrared

**Fig. 3:**
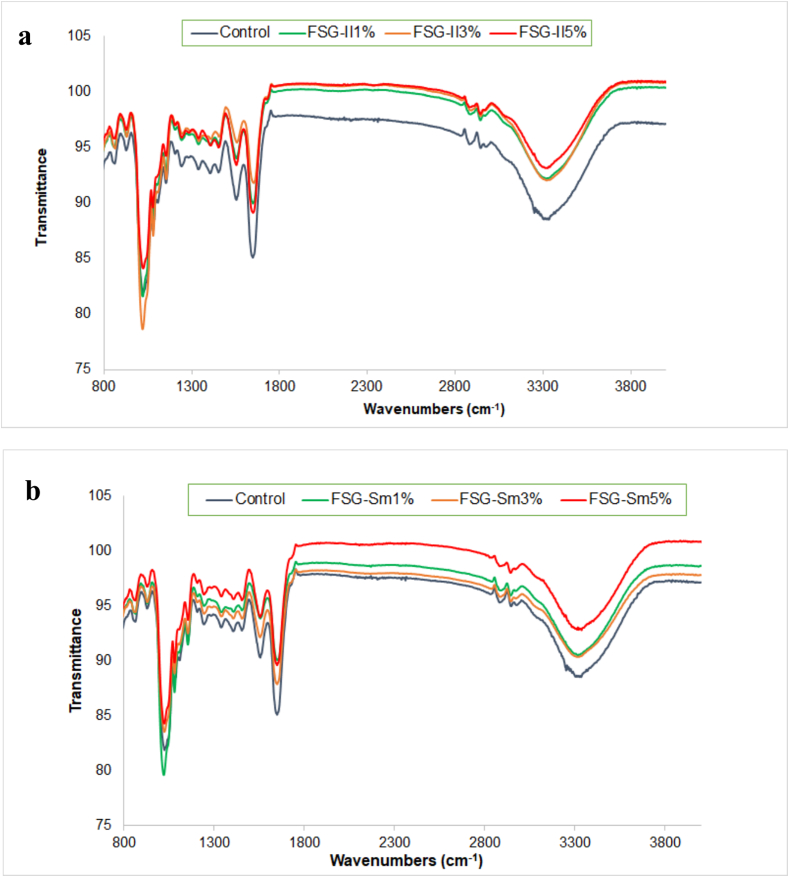
Fourier transform infrared spectroscopy patterns of gelatin nanocomposite. films; measurements were done at 25 °C and RH 50 %. (a) Smectite/gelatin nanocomposite films and (b) Illite/gelatin nanocomposites films.

FTIR spectroscopy is employed to identify characteristic bands corresponding to specific vibrational modes of functional groups present in polymers. Additionally, these vibrational spectra provide valuable insights into the interactions between Sm/Il clays and glycerol within the composite films. The spectra of Sm-gelatin and Il-gelatin films, as shown in Fig. 3, showed distinct bands. Major bands in the nanocomposite films were observed at approximately 1556–1648 cm^−1^ (amide I), attributed to C=O stretching/hydrogen bonding coupled with C—C, and 1500–1549 cm^−1^ (amide II), associated with N—H bending vibrations and C—N stretching. Further, amide A bands appeared in the 3121–3564 cm^−1^ range, reflecting NH-stretching coupled with hydrogen bonding, while amide B bands, found between 2976 and 2902 cm^−1^, represent asymmetric stretching vibrations of = C=H and –NH₃^+^. These bands highlight interactions such as C=O stretching and hydrogen bonding within the gelatin matrix (Abdelhedi et al., 2018).

Fig. 3 illustrates that the amide I band (∼1600 cm^−1^) in gelatin films originates from C=O stretching/hydrogen bonding and COO^−^ interactions. This peak shifts to higher wavelengths (∼1649 cm^−1^) in nanocomposite films, indicating conformational changes in the secondary protein structure resulting from variations in Sm/Il concentration (Kong & Yu, 2007). These changes are attributed to interactions between the tetrahedral groups of clay and the carboxylic groups in the biopolymer, which form hydrogen bonds and alter the IR absorption of COO^−^ groups. At 1518 cm^−1^, the amide II band reflects N—H bending vibrations and C—N stretching (López-Angulo et al., 2020). Amide III bands, associated with C—N and N—H group vibrations or CH₂ groups in glycine, were detected around ∼1136 cm^−1^ (Fig. 3). Bands emerging at ∼974 cm^−1^ in clay/gelatin films are linked to C—O vibrations in glycerol and Si–O–Al stretching in Smectite/Illite, as reported by López-Angulo et al. (2020). Lastly, the amide A band at ∼3330 cm^−1^ corresponds to N—H stretching vibrations, as depicted in Fig. 3.

Furthermore, the incorporation of Sm and Il did not alter the characteristic bands of gelatin, indicating that the native gelatin structure was preserved and suggests a successful interaction between the clay layers and gelatin chains (Valencia et al., 2016). This interaction could explain the improved mechanical properties observed in the gelatin matrix, attributed to strong hydrogen bonding between the clays and gelatin. In general, the addition of clay minerals to polymer matrices has been shown to enhance key packaging properties, including thermal stability, mechanical strength, physicochemical characteristics, and barrier performance (Cheikh et al., 2022; Modi et al., 2025; Sudhakar et al., 2021). Notably, the incorporation of Sm and Il promoted interactions between the positively charged –NH groups of gelatin and the negatively charged sites on the clay structure, which result from isomorphic substitutions (e.g., Al^3+^ for Si^4+^ in tetrahedral sheets, and Mg^2+^ or Fe^2+^ for Al^3+^ in octahedral sheets of smectite) (Koutsopoulou et al., 2020). These electrostatic attractions and hydrogen bonds are further supported by exchangeable interlayer cations and the negative charge of illite's basal and edge surfaces (Shao et al., 2019). Additionally, the films showed a pH of approximately 4.5, which was below the isoelectric point of gelatin. Under these conditions, gelatin molecules had a net positive charge, which could enhance electrostatic interactions with the negatively charged clay components (Goudie et al., 2023).

### 3.8 Surface properties of films

The surface properties of nanocomposite films provide critical insights into their behavior, particularly in terms of surface tension and wettability. These properties are assessed by measuring the contact angle (θ) with various solvents, with water being the most used liquid to evaluate the hydrophobic or hydrophilic nature of a film's surface.

As shown in Table 4, the water contact angle (WCA) values for the films, measured after 60 s, ranged from 72.30 ± 1.90° for the gelatin film (GF) to 126.80 ± 0.70° for GF-IL5%. According to the literature, surfaces with a WCA of approximately 65° are classified as hydrophobic (Wang et al., 2022a, 2022b), and values around 90° Karbowiak et al. (2006) further indicate hydrophobic characteristics. Based on these findings, the studied films can be classified as nearly hydrophobic. The control film had the lowest value of WCA (72.30 ± 1.90°), which tend to be increased as Sm/Ile percentage increase to reach 108.80° and 126.80° in GF-Sm5% and GF-IL5%, respectively. These analyses are comparable to those reported by Nur Amila Najwa et al. (2020) proving that the combination of gelatin and Silver-Kaolin give more resistant nanocomposite films with less accessibility to water.

**Table 4:** Surface properties of films.

	WCA (t60)	ɣ_c (mN/m)_	ɣ_S_^P^_(mN/m)_	ɣ_S_^D^_(mN/m)_	ɣ_S (mN/m)_
GF	72.30 ± 1.90 ^f^	40.2 ± 0.30 ^d^	0.17 ± 0.03 ^g^	38.15 ± 1.68 ^d^	38.32 ± 1.62 ^e^
GF-Sm1%	91.75 ± 0.40 ^e^	41.00 ± 2.35 ^c^	0.70 ± 0.16 ^f^	40.50 ± 0.60 ^d^	41.20 ± 0.80 ^d^
GF-Sm3%	93.00 ± 0.70 ^d^	38.00 ± 1.00 ^e^	4.00 ± 0.20 ^c^	46.70 ± 0.50 ^c^	50.70 ± 0.70 ^c^
GF-Sm5%	108.80 ± 1.20 ^b^	43.00 ± 0.10 ^b^	10.00 ± 0.07 ^a^	53.00 ± 0.70 ^b^	63.00 ± 0.60 ^b^
GF-IL1%	93.30 ± 1.25 ^d^	36.00 ± 1.00 ^e^	1.45 ± 0.30 ^e^	39.40 ± 1.26 ^d^	40.85 ± 1.48 ^de^
GF-IL3%	99.60 ± 0.40 ^c^	36.00 ± 0.40 ^e^	2.10 ± 0.14 ^d^	41.10 ± 0.60 ^d^	43.2 ± 0.70 ^d^
GF-IL5%	126.80 ± 0.70 ^a^	45.00 ± 0.10 ^a^	7.30 ± 0.20 ^b^	58.70 ± 0.30 ^a^	66.00 ± 0.07 ^a^

GF indicates gelatin film. GF-Sm1%, GF-Sm3%, GF-Sm5%, GF-IL 1 %, GF-IL3 %, GF-IL5 % indicate gelatin film added by 1 %, 3% and 5 % of smectite and illite, respectively. All measurements were performed at 25 °C and RH = 50 %. ^a,b,c^ Different letters in the same column indicate significant difference (*p* < 0.05).

WCA: water contact angle (t60) was measured after 60 s. Results are the mean of 5 random positions in the film; **ɣ**_**c**_: Critical surface tension; **ɣ**_**S**_^**P**^: Polar component;**ɣ**_**S**_^**D**^: Dispersive component;**ɣ**_**S**_: Total surface energy of the film (mN/m).

**Fig. 4:**
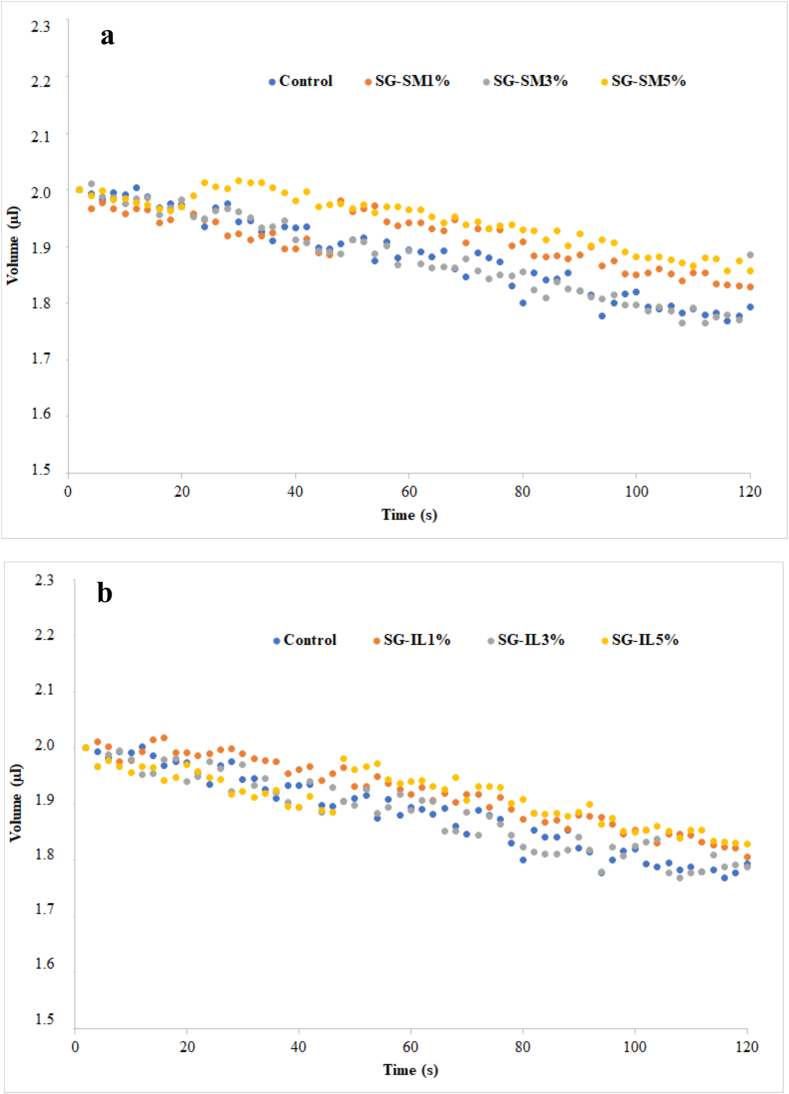
Kinetics of water droplets volume deposited on the surface of films as a function of time; measurements were done at 25 °C and RH 50 %(a) Smectite/gelatin nanocomposite films and (b) Illite/gelatin nanocomposites films.

The wettability of the films was assessed by observing the behavior of water droplets placed on the solid surface over time. Fig. 4 illustrates the kinetics of water droplet volume changes on the surface of clay/gelatin films during a 120-s period. In all samples, the droplet volume showed a slight decrease over time, primarily due to the evaporation of the solvent into an atmosphere that was not saturated with liquid vapor (Pulla-Huillca et al., 2021). In addition to water, other liquids were also used, and their solid contact angles were measured to determine the surface energy of the films, as outlined in Table 4. The results revealed a high dispersive component of surface tension (γSD), which reached values of 53.00 ± 0.70 mN/m for GF-Sm5 % and 58.70 ± 0.30 mN/m for GF-IL5 %, further confirming the hydrophobic nature of the nanocomposite films.

Along all films, the total surface energy(γ_S_) increased with the increase of dispersive component (γ_S_^D^).To note, Il at a ratio of 5 % give more hydrophobic films with γ_S_^D^ = 58.70 ± 0.30°and γ_S_ = 66.00 ± 0.07°. However, the incorporation of SM and Il at 5 % increased the polar component (γ_S_^P^) values to 10.00 ± 0.07° and 7.30 ± 0.20°, respectively, compared to the control film (0.17 ± 0.03°). In, the same context, Pulla-Huillca et al. (2021) demonstrated that the interaction between Montmorillonite and gelatin reduced γ_S_^P^ values and increased the γ_S_^D^ values.

Wettability of clay/gelatin nanocomposite films was also estimated through their critical surface tension of wetting (γ_C_) by plotting cos (θ)against (γ_L_) of the different liquids. The analysis presented in Table 4 demonstrated that the highest critical surface tension (γ_C_) for GF-IL5 % (45.00 ± 0.10) and proved that the critical tensions(γ_C_) calculated based on ethylene glycol, glycerol, water, and diiodomethane were higher to the values of γ_s_ of films. Almost, the high value of (γ_C_) reflected the hydrophobicity of nanocomposite films.

### 3.9 Scanning electron microscopy (SEM)

**Fig. 5:**
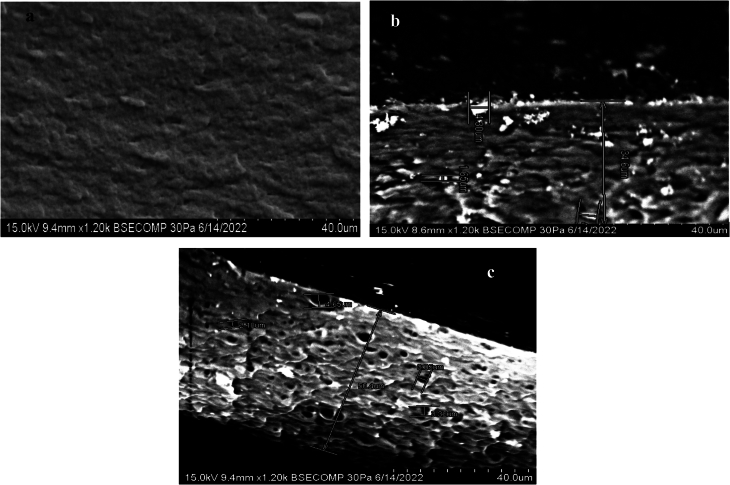
Scanning electron microscopy images of nanocomposites films (a) gelatin film, (b) Smectite/gelatin nanocomposite film and (b) Illite/gelatin nanocomposites film.

Fig. 5 shows the microstructure of clay/gelatin films. SEM scans displayed homogeneous microstructure. In fact, the low water vapor permeability and the high solubility of films sample proved that gelatin macromolecules were connected by weak bonds such as hydrogen bonds (Méité et al., 2021). FG-SM5 % micrograph revealed some small pores that contributed to the surface being more porous. This may be explained by the weak cohesion between gelatin and clay particles. Whereas, FG-Il 5% image showed a decrease of pores and a uniform distribution of clay/gelatin which demonstrated that the microstructure become denser without agglomeration suggesting a high interaction between gelatin and clay nanoparticles (Nur Amila Najwa et al., 2020).

### 3.10 Biological activities (in vitro) and biodegradability of composite films

#### 3.10.1 Antibacterial activity

The antibacterial activity of Sm-gelatin and Il-gelatin films was evaluated using the agar diffusion method against both Gram-positive and Gram-negative bacteria (Table 5). The control film GF showed no inhibitory activity against the bacterial strains, indicating that the gelatin possessed a low antibacterial effect against these pathogenic strains. However, Sm-gelatin and Il-gelatin films showed an important antibacterial activity against different bacteria as a function of Sm and Il content. The film with minimum Sm (1 %) showed a significant antibacterial activity against the four-bacteria *Staphylococcus aureus*, *Bacillus cereus*, *E. coli* and *Pseudomonas*. Hence, Table 5 showed that films with high Sm and Il content were capable to destroy the gram-positive bacteria's thicker layer, simultaneously, at the same dosage was suitable to break the thin layers of peptidoglycan corresponding to the gram-negative bacterial cells. Thus, the inhibitory activities zone diameter (IZD) of Sm and Il indicated that the film consisting of 5 % Sm showed the highest antibacterial activity (Table 5) and the IZD became more important regardless of the concentration, ranged from 12 to 19 mm for *Staphylococcus aureus*, proving the efficiency of Sm to inhibit gram positive than gram negative due to the negatively charged of Sm layers (Czímerová et al., 2006), which interacted well with the positive cell wall membrane of the gram-positive bacteria (Rangaraj et al., 2022) via an electrostatic interaction manning the rupture of cell wall and a microbial death. These results were compared with those of Najwa, et al., (2020), who reported that after kaolin modification with silver particles, the composite gelatin‑silver-kaolin films was able to destroy well wall of *Staphylococcus aureus* and to increase the inhibitory zone of *E. coli* and *S. typhimurium*. In the same context, Rangaraj et al. (2022) reported that silver sepiolite-gelatin films enriched with date waste extract is able to inhibit the antibacterial activity against both gram-positive and gram-negative microbes (*E. coli* and *S. aureus*).

**Table 5:** Antibacterial activity of smectite-gelatin and illite-gelatin films.

	*B. cereus*	*P. aeruginosa*	*S. aureus*	*E. coli*
GF-Sm1%	12.00 ± 0.50 ^d^	11.50 ± 1.0 ^c^	6.00 ± 0.5 ^a^	9.00 ± 0.50 ^b^
GF-Sm3%	15.10 ± 0.33 ^b^	13.5 ± 1.0 ^b^	7.25 ± 0.5 ^c^	9.50 ± 0.50 ^b^
GF-Sm5 %	19.10 ± 0.5 ^a^	17.00 ± 1.0 ^a^	10.0 ± 0.5 ^a^	17.20 ± 1.0 ^a^
GF-IL1%	11.00 ± 0.5 ^d^	8.50 ± 1.0 ^e^	8.50 ± 0.5 ^b^	8.00 ± 1.0 ^c^
GF-IL3%	14.20 ± 0.5 ^c^	10.30 ± 0.5 ^d^	7.50 ± 1.0 ^c^	5.00 ± 1.0 ^e^
GF-IL5 %	15.60 ± 0.5 ^b^	12.30 ± 0.5 ^b^	8.50 ± 0.5 ^b^	7.5 ± 1.0 ^d^

GF indicates gelatin film. SG-Sm1 %, SG-Sm3%, SG-Sm5 % indicate gelatin film added by 1 %, 3% and 5 % of smectite, GF-IL 1 %, GF-IL3%, GF-IL5 % respectively. All measurements were performed at 25 °C and RH = 50 %. ^a,b,c^ Different letters in the same column indicate significant difference (*p* < 0.05).

Overall, the observed antibacterial activity of Sm and Il films is attributed to their ion-exchange capacity and high surface area, indicating their potential as active materials in antimicrobial food packaging applications.

#### 3.10.2 Antioxidant activities of clays-gelatin films

**Fig. 6:**
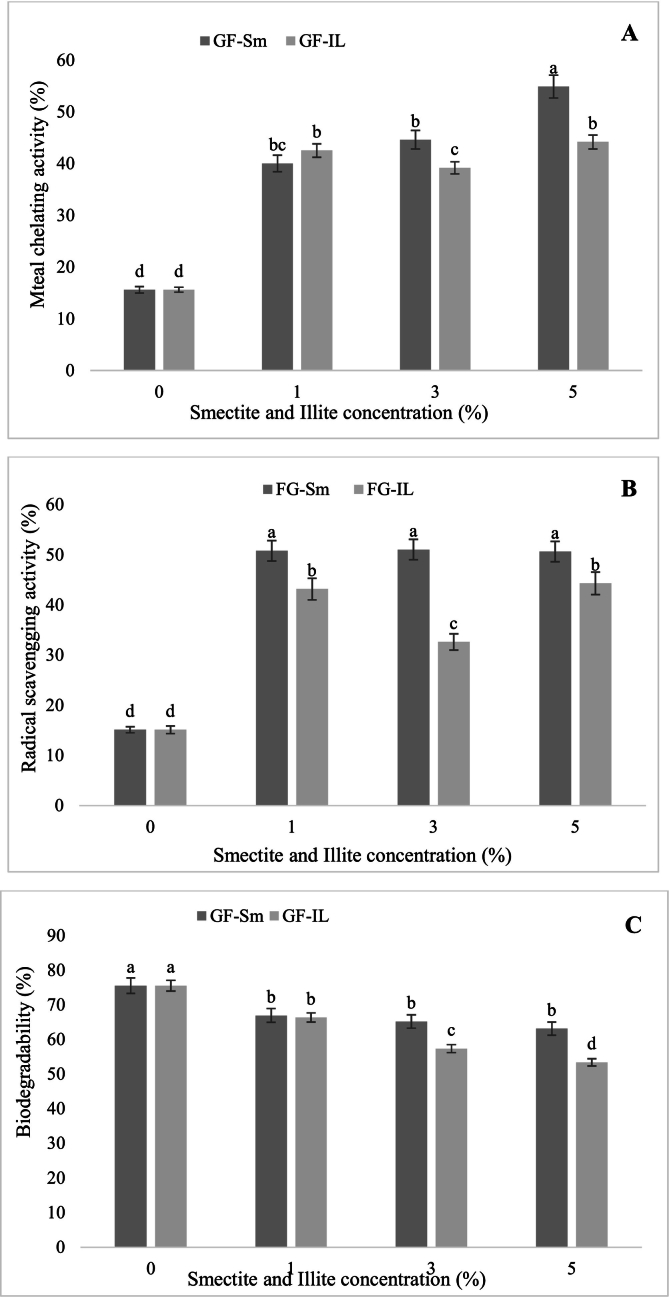
(A) Metal-chelating activity; (B) Free radical scavenging activity on 1,1-diphenyl-2-picrylhydrazyl (DPPH•) and (C) biodegradability (%) of clay–gelatin films.

Antioxidant activity of Sm-gelatin and Il-gelatin films was evaluated using DPPH scavenging activity (Fig. 6A) and metal-chelating activity (Fig. 6B). The obtained results showed that control film GF had a lower antioxidant activity compared to those added with Sm and Il regardless the concentrations. The addition of Sm and Il gelatin films increased the antioxidant power when compared to gelatin film. In fact, GF-Sm5% and FG-IL5% showed the highest DPPH-radical scavenging to reach a value of 50 and 45 %. The antioxidant activity of the composite film is mainly due to the enhancement of agent activity. Moreover, the outer surface of clays is negatively charged due to Si–O–Si bonds (Massaro et al., 2020). This structure could cause chemically positively and negatively charged ions to be attached inside or outside the clays, respectively. Meanwhile, the positive charge of inner surface provides a large area for negative charge of free radicals to be attached (Çankaya et al., 2024). It was proposed that clays act as a barrier to the transport of low molecular weight components due to it impermeable properties. Moreover, the results presented in Fig. 6B showed that Sm- and Il-gelatin films exhibited ferrous ion (Fe^2+^) chelating activity. The chelating capacity of Sm-gelatin films increased significantly with rising Sm concentrations, with the GF-Sm5% film showing the highest activity (*p* < 0.05). This enhanced chelation is likely attributed to the high surface area and ion-exchange capacity of Sm, which facilitated effective binding of metal ions.

#### 3.10.3 Biodegradation test of clays-gelatin films

The biodegradation of nanocomposite films was employed to estimate the natural degradation conditions and to expose films to microorganism action. Regarding the biodegradation test of nanocomposite films (Fig. 6C), the Sm-gelatin and Il-gelatin films revealed 63 % and 53 % weight loss after 10 days, proving moderate biodegradability of films. Furthermore, Fig. 5C showed that FWL depends on time contact with soil. In this study, a close relationship between mechanical properties and biodegradation results confirmed that clays enhanced the rigidity of films. These findings explained the higher degradation of control film (75 %) compared with Sm-gelatin and Il-gelatin films. These results were compared with those of Chang et al. (2022) who reported that gelatin film degradation after 18 days and proved great biodegradability in dry soil. Several studies reported that films containing organic components are highly susceptible to biodegradation. In the case of gelatin-based films, the high weight loss is primarily attributed to microbial activity targeting the amorphous regions of the polymer chains. Consequently, the biodegradation rate can reach 100 % (Abarca et al., 2022; de Oliveira Filho et al., 2019).

### 3.11 Characterization of coated cherry fruits

**Fig. 7:**
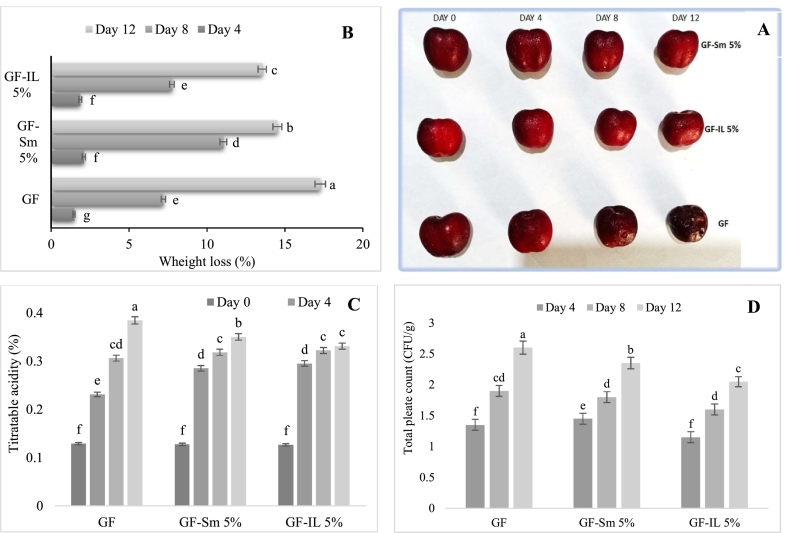
Weight loss (A), illustration (B), titratable acidity (C) and microbial count of cherry fruits during storage.

The effectiveness of the coating formulations (GF-Sm5 % and GF-IL5 %) on cherry fruits was evaluated throughout the storage period (Fig. 7A). The results emphasize key quality parameters, including weight loss, titratable acidity, color parameters and microbial stability, to assess the performance of each treatment.

#### 3.11.1 Weight loss

The weight loss test serves as a key indicator of dehydration over the storage period, with excessive weight loss signifying inadequate preservation (Lufu et al., 2020). To evaluate the effectiveness of various gelatin films in maintaining cherry freshness, the weight loss of packaged cherries were evaluated (Fig. 7B). The results indicated that all coating solutions effectively preserved fruit quality parameters throughout storage when compared to the control. Among them, cherries coated with GF-Il5% exhibited the most pronounced delay in weight loss, ranging from 1.9 to 13.54 %, suggesting enhanced moisture retention. Similarly, GF-Sm5% contributed to delay dehydration, with a weight loss range of 2.1 to 14.5%, reinforcing its role in maintaining fruit integrity over time. In the same context, Zhang et al. (2024) reported that PLA at 8 mmol/L effectively preserved cherry quality by reducing weight loss and preventing spoilage. Another study by Crescente et al. (2024) reported that PLA films with micro-perforation significantly reduced weight loss in strawberries. Moreover, Li et al. (2025) demonstrated the effective use of composite films made from soybean protein isolate and gelatin, incorporated with bentonite and enriched with rosemary, in preserving fresh lemon slices by delaying mold formation and limiting weight loss to 37.89 %

#### 3.11.2 Titratable acidity (TA)

The acidity of fruits plays an important role in defining their flavor profile, as it is influenced by the presence of organic acids such as malic, citric, and tartaric acid (Shi et al., 2022). Given that malic acid is the predominant organic acid in cherries, it was selected as the key parameter for analyzing TA in this study and results were presented in Fig. 7C. A decline in TA was observed in both coated and control fruits throughout storage. However, the coated fruits showed a slow decrease, indicating enhanced preservation of acidity. Among the coatings, GF-Sm5% and GF-IL5% effectively delayed the reduction in TA, with a noticeable impact by the 12^th^ day of storage, suggesting their role in maintaining the fruit's organic acid balance for an extended period. Similar trends have been observed with PLA films for cherry packaging (Hu et al., 2023; Soltani et al., 2025; Zhang et al., 2024).

#### 3.11.3 Color parameters

Color is a key quality indicator for fruit freshness and overall appearance. Table 6 presented the changes in surface color parameters of cherry fruits over a 12-day storage period, illustrating variations in L*, a*, b*, C, and *h* values. The higher values observed in uncoated cherries suggested an increased tendency toward browning, greater redness, yellowness, chromatic intensity, and hue shifts compared to coated cherries. The accelerated discoloration in uncoated samples is likely attributed to oxidation, ripening processes, and degradation mechanisms occurring at a faster rate, emphasizing the protective role of coating treatments in maintaining color stability over time (Wani et al., 2021). The obtained results are aligned with previous studies that confirmed that PLA films helped to maintain polyphenol content and color stability in strawberries and cherries (Hu et al., 2023; Soltani et al., 2025; Wani et al., 2021; Zhang et al., 2024).

**Table 6:** Color test of smectite-gelatin and illite-gelatin films.

		Storage Day			
	Treatment	0	4	8	12
L* value	GF	36.2 ± 0.20 ^b^	35.83 ± 0.90 ^b^	32.6 ± 0.30 ^b^	27.1 ± 0.44 ^b^
	GF-Sm 5 %	25.67 ± 0.10 ^c^	30.3 ± 0.20 ^c^	34.4 ± 0.30 ^a^	34.9 ± 0.90 ^a^
	GF-IL 5 %	38.67 ± 0.50 ^a^	37.04 ± 0.83 ^a^	35.0 ± 0.02 ^a^	35.6 ± 0.60 ^a^
a* value	Control	16.2 ± 0.32 ^a^	17 ± 0.16 ^a^	20 ± 0.60 ^b^	26.9 ± 0.60 ^a^
	GF	14.7 ± 0.35 ^c^	18 ± 0.30 ^a^	24 ± 0.12 ^a^	24.3 ± 0.70 ^b^
	GF-Sm 5 %	15.8 ± 0.20 ^b^	17.3 ± 0.25 ^a^	19 ± 0.34 ^b^	23 ± 0.31 ^c^
	GF-IL 5 %	17.2 ± 0.45 ^a^	17.4 ± 0.75 ^a^	21.1 ± 0.42 ^a^	22.8 ± 0.30^d^
b* value	GF	4.3 ± 0.60 ^b^	8.7 ± 0.90 ^a^	13.4 ± 0.50 ^a^	14.7 ± 0.35 ^a^
	GF-Sm 5 %	8.1 ± 0.30 ^a^	8.5 ± 0.40 ^a^	8.5 ± 0.10 ^b^	8.8 ± 0.15 ^b^
	GF-IL 5 %	4.6 ± 0.60 ^b^	7.4 ± 0.26 ^a^	7.8 ± 0.12 ^b^	9.4 ± 0.20 ^b^
C* value	GF	14.5 ± 0.42 ^b^	16.8 ± 0.38 ^c^	19.3 ± 0.20 ^a^	21.3 ± 0.80 ^b^
	GF-Sm 5 %	16.5 ± 0.10 ^b^	20.1 ± 3.8 ^a^	20.3 ± 4.5 ^a^	21.1 ± 0.36 ^b^
	GF-IL 5 %	18.3 ± 0.30 ^a^	18.9 ± 0.90 ^b^	20.3 ± 0.95 ^a^	24.1 ± 0.44 ^a^
h value	GF	20.9 ± 0.80 ^a^	20.1 ± 0.75 ^a^	16.5 ± 0.22 ^b^	14 ± 0.30 ^c^
	GF-Sm 5 %	16.8 ± 0.50 ^b^	18.9 ± 0.09 ^b^	19.9 ± 0.90 ^a^	23.6 ± 0.60 ^b^
	GF-IL 5 %	17.8 ± 0.30 ^b^	19.6 ± 0.7 ^ab^	21.1 ± 0.60 ^a^	26.6 ± 0.50 ^a^
ΔE	GF	7.98 ± 0.10 ^b^	9.35 ± 0.95 ^a^	5.32 ± 0.50 ^a^	4.19 ± 0.52 ^b^
	GF-Sm 5%	12.35 ± 0.90 ^a^	8.71 ± 0.50 ^ab^	2.07 ± 0.50 ^b^	8.90 ± 0.60 ^a^
	GF-IL 5%	13.03 ± 0.70 ^a^	7.11 ± 0.94 ^b^	0.92 ± 0.50 ^c^	4.14 ± 0.80 ^b^

GF indicates gelatin film. SG-Sm1%, SG-Sm3%, SG-Sm5% indicate gelatin film added by 1 %, 3% and 5% of smectite, GF-IL 1%, GF-IL3%, GF-IL5% respectively. All measurements were performed at 25 °C and RH = 50 %. ^a,b,c^ Different letters in the same column within the same parameter indicate significant difference (*p* < 0.05).

#### 3.11.4 Microbiological quality

The Fig. 7D illustrated the psychrophilic bacteria, yeast, and mold count determined in both coated and uncoated cherries fruits during 12 days of refrigeration at 4 °C. The results indicated that coating treatments effectively reduced psychrophilic bacterial growth compared to the uncoated control. This reduction suggested that the coatings provided a protective barrier, limiting microbial proliferation by reducing direct exposure to oxygen and moisture, which are critical factors in microbial development (Karnwal et al., 2025). Additionally, the coatings inhibited the enzymatic activity or prevented adhesion of spoilage microorganisms, contributing to extended microbial stability throughout storage. The observed differences between coated and uncoated fruits further emphasize the role of these films in preserving fruit quality and safety during cold storage (Karnwal et al., 2025).

## 4 Conclusion

This study showed that gelatin nanocomposite films reinforced with smectite and illite clays effectively enhanced mechanical, thermal, and barrier properties, while providing antimicrobial and antioxidant benefits. These improvements made the films particularly suitable for coating and preserving cherry fruits, helping to maintain quality and extend shelf life through reduced spoilage and oxidation. Thus, these biodegradable films represented a sustainable and functional packaging for fresh cherries preservation.

## CRediT authorship contribution statement

**Nermine Sayah:** Methodology, Formal analysis, Conceptualization. **Nasir A. Ibrahim:** Resources, Project administration, Methodology, Investigation, Formal analysis. **Walid Elfalleh:** Writing – review & editing, Visualization, Validation. **Nacim Zouari:** Writing – review & editing, Supervision. **Noureddine Hamdi:** Project administration. **Mourad Jridi:** Writing – review & editing, Writing – original draft, Visualization, Software, Methodology, Investigation.

## Funding statement

This work was supported and funded by the Deanship of Scientific Research at Imam Mohammad Ibn Saud Islamic University (IMSIU) (grant number IMSIU-DDRSP-RP25).

## Declaration of competing interest

The authors declare that they have no known competing financial interests or personal relationships that could have appeared to influence the work reported in this paper.

## Data Availability

Data will be made available on request.
